# More anterior bone loss in middle vertebra after contiguous two-segment cervical disc arthroplasty

**DOI:** 10.1186/s13018-024-04663-6

**Published:** 2024-04-12

**Authors:** Minghe Yao, Tingkui Wu, Hao Liu, Kangkang Huang, Junbo He, Shihao Chen, Beiyu Wang

**Affiliations:** 1 Department of Orthopedics, West China Hospital, Sichuan University, 37 Guoxue Lane, Chengdu, Sichuan 610041 China; 2 Department of Orthopedics, Orthopedic Research Institute, West China Hospital, Sichuan University, 37 Guoxue Lane, Chengdu, Sichuan 610041 China

**Keywords:** Cervical disc arthroplasty, Vertebral body, Sagittal area, Anterior bone loss, Heterotopic ossification

## Abstract

**Background:**

Contiguous two-segment cervical disc arthroplasty (CDA) is safe and effective, while post-operative radiographic change is poorly understood. We aimed to clarify the morphological change of the three vertebral bodies operated on.

**Methods:**

Patients admitted between 2015 and 2020 underwent contiguous two-level Prestige LP CDA were included. The follow-up was divided into immediate post-operation (≤ 1 week), early (≤ 6 months), and last follow-up (≥ 12 months). Clinical outcomes were measured by Japanese Orthopedic Association (JOA) score, visual analogue score (VAS), and neck disability index (NDI). Radiographic parameters on lateral radiographs included sagittal area, anterior-posterior diameters (superior, inferior endplate length, and waist length), and anterior and posterior heights. Sagittal parameters included disc angle, Cobb angle, range of motion, T1 slope, and C2-C7 sagittal vertical axis. Heterotopic ossification (HO) and anterior bone loss (ABL) were recorded.

**Results:**

78 patients were included. Clinical outcomes significantly improved. Of the three operation-related vertebrae, only middle vertebra decreased significantly in sagittal area at early follow-up. The four endplates that directly meet implants experienced significant early loss in length. Sagittal parameters were kept within an acceptable range. Both segments had a higher class of HO at last follow-up. More ABL happened to middle vertebra. The incidence and degree of ABL were higher for the endplates on middle vertebra only at early follow-up.

**Conclusion:**

Our findings indicated that after contiguous two-segment CDA, middle vertebra had a distinguishing morphological changing pattern that could be due to ABL, which deserves careful consideration before and during surgery.

**Supplementary Information:**

The online version contains supplementary material available at 10.1186/s13018-024-04663-6.

Cervical disc arthroplasty (CDA) is a non-fusion and movement-preserving intervertebral technique [1]. As a treatment for cervical spondylosis, clinical studies have confirmed that multi-segment CDA is equally effective and safe compared to single-segment CDA [2, 3]. However, it is important to note that there are significant differences between the two approaches, extending beyond simply the number of segments. Involved in single-segment CDA, two vertebrae are operated on adjacent to only one artificial disc, whereas contiguous two-segment CDA involves the clamping of a middle vertebra, with both endplates operated on and meeting the implanted discs. Related to implantation, the vertebral body often experiences various morphological alternations including anterior bone loss (ABL) [4] and heterotopic ossification (HO) [5]. Therefore, middle vertebral body may undergo a change different from the non-middle ones, which has not been fully elucidated. The present study aimed to measure the morphological changes of the operated vertebrae after contiguous two-segment CDA.

## Materials and methods

We reviewed the records of patients treated at our institution from January 2015 to December 2020 undergone contiguous two-level Prestige LP CDA procedures. Ethical approval was given by the medical ethics committee of our institution. Post-operative follow-up was divided into three periods: immediate period (within 1 week post-operatively), early period (within 6 months), and last follow-up (12 months and beyond). All included patients should attend all three periods of follow-up. When multiple records were available in one follow-up period, only the latest record was taken.

Patient demographics, clinical outcomes, and radiographic parameters were collected. Data were collected pre-operatively and during the three post-operative follow-up periods.

Clinical outcomes were assessed using the Japanese Orthopedic Association (JOA) cervical spine function score, visual analogue score (VAS) for neck pain, and neck disability index (NDI).

**Fig. 1:**
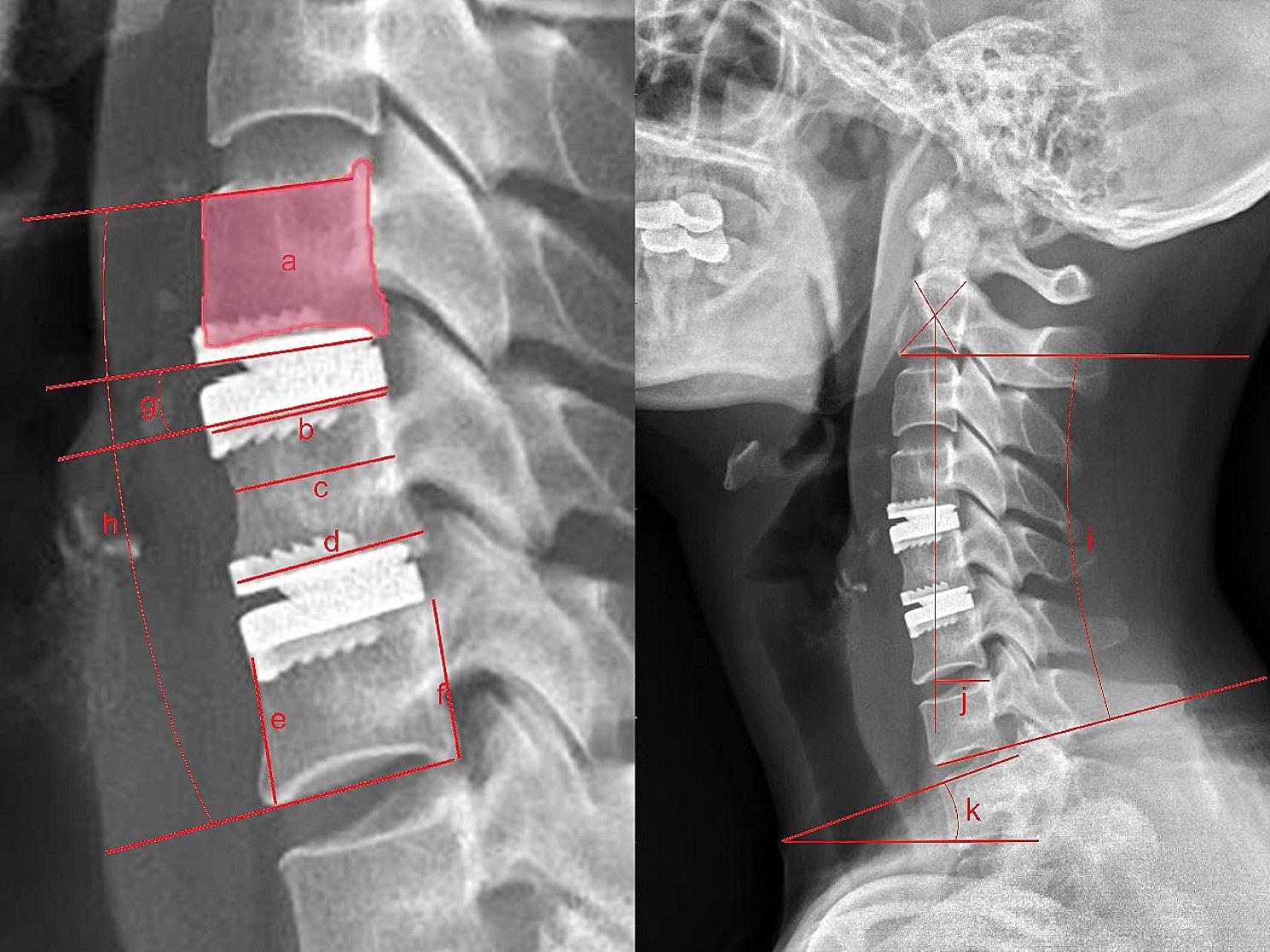
Schematic diagram of imaging measurements. **a**. Sagittal area; **b/c/d**. Superior endplate/middle vertebral/inferior endplate length; **e/f**. Anterior/posterior height; **g**. Disc angle; **h**. Angle of surgery levels; **i**. Cobb angle of C27; **j**. C2-C7 sagittal vertical axis; **k**. T1 slope

Imaging evaluation (Fig. 1) included sagittal area, anterior-posterior diameters (superior and inferior endplate lengths and waist length), anterior and posterior heights, disc angle (DA), Cobb angle of C2-C7 (CobbC27), angle of surgery levels, ROM of C2-C7 (ROMC27), ROM of surgery levels (ROMSL), T1 slope (T1S), C2-C7 sagittal vertical axis (SVA), HO, and ABL. All parameters were measured on lateral radiographs. Among them, sagittal area was defined as the area of the region enclosed by cortical bone of vertebral body. Anterior-posterior diameter was defined as the distance between anterior and posterior margins of vertebral body, of which superior and inferior endplate length was measured at endplate, and the waist length was measured in between and at the narrowest place. The anterior and posterior heights were defined as the distance between superior and inferior margins of vertebral body and were measured at anterior and posterior margins. DA, CobbC27, ROMC27, ROMSL, T1S and SVA were measured as previously reported [6–8]. HO was graded according to McAfee et al. from grade 0 to grade IV [9] and was classified into low-grade (grade 0, I, and II) and high-grade (grade III and IV). ABL was measured and categorized as Wu et al [10], which was based on Kieser et al. [11]. ABL incidence of vertebra was calculated by each endplate. All measurements were performed on radiographs using ACDsee Canvas 14 (ACD Systems, Canada) and ImageJ 1.52 (National Institutes of Health, USA). To eliminate the magnification effect of the radiographs, we converted the length and area measurements to actual values by a method reported [12]. Continuous data were expressed as mean and standard deviation (± SD). Categorical data were as numbers and percentages.

SPSS 22.0 software was used for statistical analysis. Comparisons of HO and ABL were performed using chi-square test (for incidence rate and two-class grading) or Mann-Whitney U test (for ordered multi-class grading). Comparisons of clinical outcomes and the other radiographic parameters used one-way repeated-measures ANOVA. Pairwise comparisons were conducted for variables showing significant differences, with multiple comparisons adjusted using the Bonferroni method. Statistical significance was defined as a two-sided p-value of less than 0.05.

## Results

According to the criteria, a total of 78 patients including 38 men and 40 women completed follow-up in this study, with a mean follow-up of 47.8 months (at least 12 months). The mean age at the time of surgery was 44.7 years. The mean blood loss was 84.6 mL. Implantation levels included eight C3/4 and C4/5 (10.3%), 50 C4/5 and C5/6 (64.1%), and 20 C5/6 and C6/7 (25.6%) (Table 1).

**Table 1:** Demographic and surgical data of patients

Variables	Value
No. of patients (n)	78
Gender (M/F)	38/40
Age (years)	44.7 ± 8.9
BMI (kg/m^2^)	24.1 ± 2.7
BMD T value (spine)	0.9 ± 1.1
BMD (spine, g/cm^2^ )	1.2 ± 0.1
Osteopenia (Y/N)	3/75
Surgical segment	
C3/4 and C4/5	8
C4/5 and C5/6	50
C5/6 and C6/7	20
Operation time (minutes)	169.4 ± 33.1
Blood loss (mL)	84.6 ± 44.1
Follow-up time (months)	47.8 ± 30.8

BMI, body mass index; BMD, bone mineral density

### Clinical outcomes

Mean JOA, NDI, and VAS scores improved significantly post-operatively and were kept throughout the follow-up period (*p* < 0.05) (Supplementary Table 1).

### Radiographic outcomes

#### Sagittal area

**Fig. 2:**
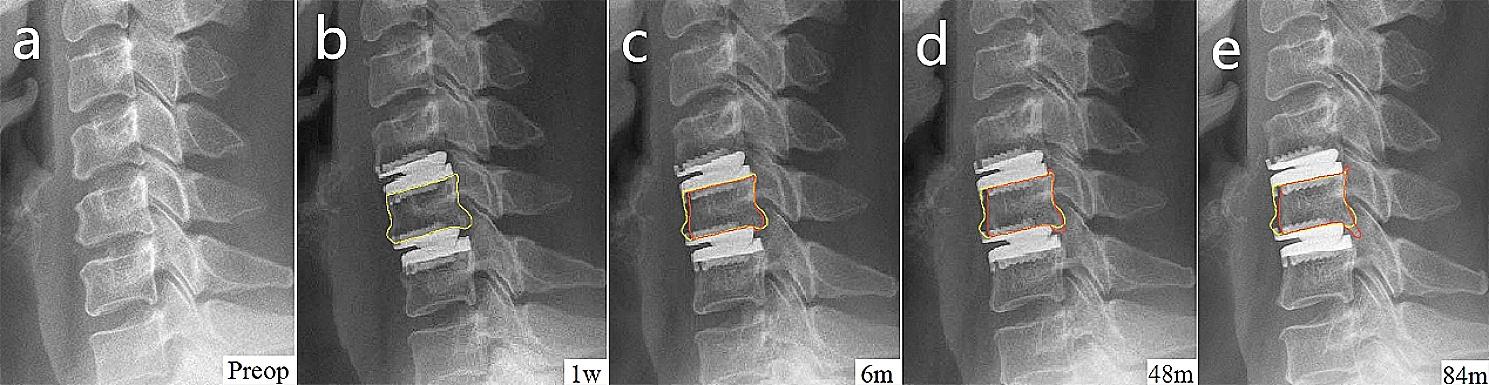
Serial radiographs of a representative case underwent contiguous two-level (C4/5 and C5/6) cervical disc arthroplasty. The series showed the sagittal area loss of middle vertebra. The changes in middle vertebral body profile are highlighted, with a yellow outline indicating the reference profile of 1-week post-operative and red outlines indicating the profile of each follow-up thereafter. **a** Pre-operative lateral radiograph of the cervical spine; **b** 1-week post-operative lateral radiograph showed cervical disc arthroplasty at C4/5 and C5/6 levels; **c** Early follow-up lateral radiograph showed moderate ABL at the anterior part of middle vertebral body; **d** and **e** Late follow-up lateral radiographs showed no obvious progress of anterior bone loss, but high-class heterotopic ossification developed at the posterior edge of caudal endplate. Sagittal area of C5 (follow-up time): 181mm^2^ (1 week), 172mm^2^ (6 months), 178mm^2^ (48 months), 179mm^2^ (84 months)

**Fig. 3:**
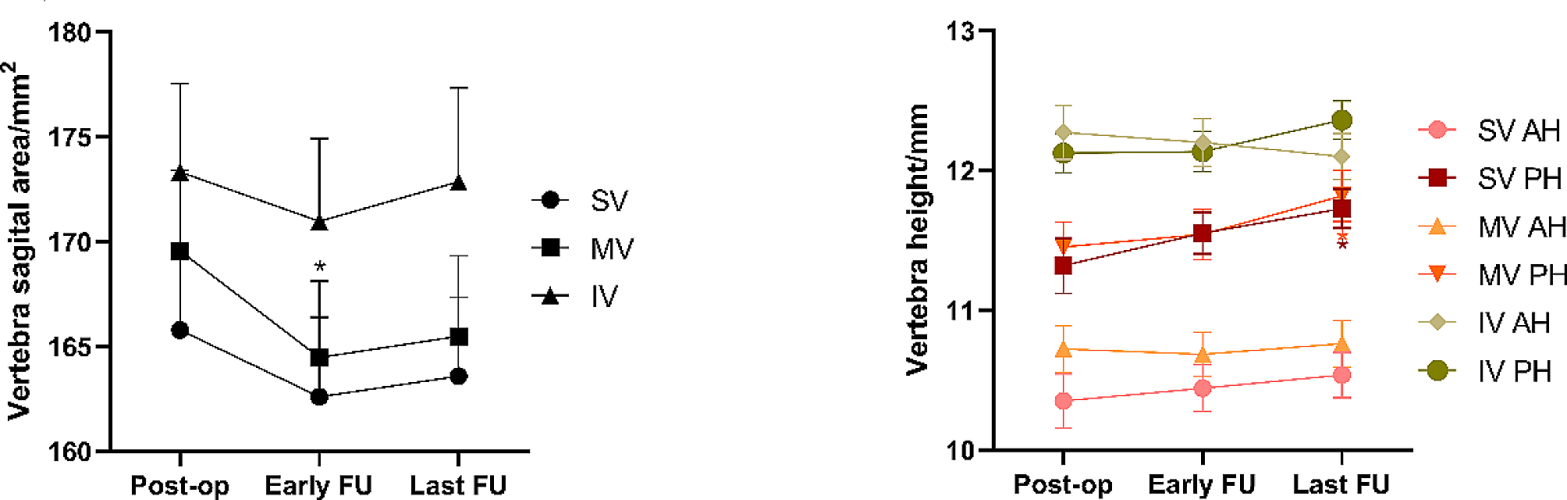
Sagittal area of vertebra,and anterior and posterior height of vertebra after surgery SV, superior vertebra. MV, middle vertebra. IV, inferior vertebra. AH, anterior height; PH, posterior height; Post-op, post-operation; FU, follow-up. Represented as mean and SEM. *P < 0.05, compared with post-operation in pairwise comparison

All three sagittal areas showed a trend of early decrease followed by an increase. In particular, the mean area of middle vertebral body decreased significantly to 164.5mm^2^ ± 21.9mm^2^ in the early period, and the difference was statistically significant compared with 169.6mm^2^ ± 23.1mm^2^ in the immediate post-operative period (*p* = 0.027). Changes in the areas of superior and inferior vertebral bodies were not significant (Figs. 2 and 3).

#### Anterior-posterior diameter

For superior vertebra, there was an increase but not statistically significant in the length of superior endplates. Waist length remained generally unchanged. There was a significant decrease in inferior endplate length, from immediate 15.9 mm ± 1.4 to 15.4 mm ± 1.5 mm in the early period (*p* = 0.001) and 15.2 mm ± 1.5 mm at last follow-up (*p* < 0.001).

In terms of the middle vertebra, there was a significant reduction in both the superior and inferior endplate lengths, primarily occurring early on. The immediate, early, and last follow-up measurements for the superior endplates were 15.8 mm ± 1.5 mm, 15.2 mm ± 1.6 mm (*p* = 0.003), and 15.2 mm ± 1.5 mm (*p* = 0.001) respectively; while for the inferior endplates they were 16.3 mm ± 1.4 mm, 15.6 mm ± 1.5 mm, and 15.7 mm ± 1.mm (both *p* < 0.001). In contrast, the waist length remained stable throughout.

Superior endplate length of inferior vertebral body significantly reduced from 16.2 mm ± 1.4 mm post-operatively to 15.5 mm ± 1.4 mm in the early period (*p* < 0.001). It increased slightly to 15.6 mm ± 1.5 mm lately. Waist length did not change significantly. Inferior endplate length increased at last follow-up (16.8 mm ± 1.7 mm) compared to immediate post-operative (16.3 mm ± 1.7 mm, *p* = 0.013) (Table 2).

**Table 2:** Comparison of anterior-posterior diameters within each vertebra

Vertebra	AP diameter, mm	FU period			P
		Post-operation	Early FU	Last FU	
SV	SEL	14.6 ± 1.8	14.9 ± 1.6	14.9 ± 1.8	0.076
	MVL	14.4 ± 1.5	14.5 ± 1.4	14.5 ± 1.5	0.590
	IEL	15.9 ± 1.4	15.4 ± 1.5*	15.2 ± 1.5**	< 0.001
MV	SEL	15.8 ± 1.5	15.2 ± 1.6*	15.2 ± 1.5*	< 0.001
	MVL	14.6 ± 1.5	14.7 ± 1.5	14.6 ± 1.5	0.822
	IEL	16.3 ± 1.4	15.6 ± 1.5**	15.7 ± 1.5**	< 0.001
IV	SEL	16.2 ± 1.4	15.5 ± 1.4**	15.6 ± 1.5*	< 0.001
	MVL	14.8 ± 1.4	14.9 ± 1.3	14.9 ± 1.4	0.415
	IEL	16.3 ± 1.7	16.7 ± 1.6	16.8 ± 1.7*	0.008

AP, anterior-posterior; SV, superior vertebra; MV, middle vertebra; IV, inferior vertebra; SEL, superior endplate length; MVL, middle vertebra length; IEL, inferior endplate length; FU, follow-up. *P < 0.05, compared with post-operation in pairwise comparison;** *P* < 0.001, compared with post-operation in pairwise comparison

#### Anterior and posterior heights

The height of the anterior margin of all three vertebrae had no significant change. The posterior heights of all three showed an increasing trend, and the difference of superior and middle vertebrae between last follow-up (11.7 mm ± 0.9 mm, 11.8 mm ± 1.1 mm) and the immediate post-operative period (11.3 mm ± 1.2 mm, 11.5 mm ± 1.1 mm) was significant (*p* = 0.034, *p* = 0.011) (Fig. 3).

#### CobbC27 and angle of surgery levels

Similarly, CobbC27 and angle of surgery levels both showed a decreasing trend. The decrease occurred mainly in the early period (CobbC27: from 15.3°±10.4° to 12.0°±10.2°, *p* = 0.006; angle of surgery levels: from 7.1°±8.6° to 2.8°±7.0°, *p* < 0.001) (Supplementary Fig. 1).

### ROM

ROMC27 increased substantially to 42.9°±9.5° in the early period and slightly to 43.8°±10.5° at last follow-up, compared with immediate 26.7°±10.1° (both *p* < 0.001). ROMSL increased significantly from 13.0°±8.1° to 17.5°±6.4° in the early period (*p* = 0.017) and 16.7°±7.1° at last follow-up (Supplementary Fig. 1).

### T1S

With a pre-operative 23.5°±6.8°, T1S reached a peak at 26.0°±6.4° after surgery. After an early fall back to 22.8°±7.2°, it decreased slightly to 22.5°±7.0° at last follow-up. No difference reached statistical significance compared with pre-operative (Supplementary Fig. 2).

### SVA

SVA increased from 12.2 mm ± 8.9 mm to post-operative 15.0 mm ± 7.3 mm, and after sliding to 12.7 mm ± 6.9 mm in the early period, it rebounded to 14.0 mm ± 6.5 mm at last. No difference between the follow-ups and the pre-operative was significant (Supplementary Fig. 2).

### DA

Both segments’ DAs significantly dropped. The upper disc decreased from post-operative 4.8°±4.1° to 2.1°±2.9° then 1.9°±3.1° (both *p* < 0.001). The corresponding values for the lower disc were 3.5°±3.8°, 2.1°±3.9°, and 1.9°±3.2° (*p* = 0.015, 0.013)(Supplementary Fig. 3).

### HO and ABL

At the final follow-up, HO occurred in 54 (69.2%) of 78 patients, involving 88 (56.4%) of 156 segments. High-grade HO (grade III or IV) affecting mobility presented in 46 (29.5%) surgical segments. Last HO grades were statistically significantly higher than early for both segments (*p* < 0.01) (Supplementary Table 1).

At the final follow-up, ABL was detected in 62 out of 78 patients (79.5%). Among the operated segments (156 in total), 92 were involved (60.0%), with 38 of them showing involvement on both endplates (24.4%). ABL affected a total of 114 vertebrae out of the operated-related ones (234 in total) with an incidence rate of 48.7%. Specifically, there were 26 cases involving superior vertebrae (incidence rate: 33.3%), while middle vertebrae had a higher incidence rate at 69.2% for its involvement in ABL, and inferior vertebrae showed an incidence rate of 43.6%. The incidences of ABL were significantly higher in middle vertebrae compared to superior and inferior vertebrae (*p* < 0.01) as shown in Table 3.

**Table 3:** ABL incidence of vertebrae

FU period	SV	MV	IV	P
Early FU	24 (30.8%)	48 (61.5%*)	28 (35.9%)	< 0.001
Last FU	26 (33.3%)	54 (69.2%#)	34 (43.6%)	< 0.001

FU, follow-up; ABL, anterior bone loss; SV, superior vertebra; MV, middle vertebra; IV, inferior vertebra; *, *P* < 0.05 in pairwise comparisons at early FU; #, *P* < 0.05 in pairwise comparisons at last FU.

Regarding the four endplates meeting the prosthesis, more instances of ABL occurred on the endplates located on middle vertebrae; however, this difference was only significant during early follow-up period (early follow-up: 46.2% vs. 33.3%, *p* = 0.021; last follow-up: 44.9% vs. 38.5%, *p* = 0.251). Furthermore, there was a significant difference observed regarding grading for ABL during early period as well(*p* = 0.008) as presented in Supplementary Table 3.

## Discussion

### Sagittal area changed by ABL and HO

Sagittal area changes can be covered by endplate length and the posterior height of the vertebral body. In the early period, area was mainly influenced by the reduction in endplate length. As middle vertebra had two shortened endplates, its area change was more significant than that of superior and inferior vertebrae. At last follow-up, endplates were stable, whereas the posterior height of all three vertebrae increased, which may represent HO formation (taking into account the high prevalence of HO and its progression at last follow-up) and consequently led to an increase in the area (Fig. 2).

### Anterior-posterior diameter and ABL

The anterior-posterior diameters can be classified into three groups: (1) For the four endplates that meet the artificial disc (e.g., inferior endplate of superior vertebra), their lengths significantly decreased early and remained static after. (2) The two endplates that do not meet the disc (e.g., superior endplate of superior vertebra) had a tendency to grow in length. (3) No significant change in waist lengths.

For group (1), the reduction in length can be explained by ABL. ABL was present in 60% of the segments in this study, and in approximately 25% of the segments, ABL involved both endplates. The change in group (1) is consistent with the bone resorption observed in previous studies on CDA [10, 13, 14] and is considered to be an adaption of bone to a new biomechanical environment [15]. According to Frost’s theory, resorption will occur to reduce the amount of unwanted bone when the functional load applied to the bone does not reach the desire [16]. A finite element analysis showed that the posterior part of the Prestige LP carried more pressure, which may imply a redistribution of pressures on endplate after implantation [17]. Group (3) did not seem to be involved in this process. Inside the vertebral body, superior endplate pressure may converge during downward conduction, pass through the waist region, and redistribute eventually to inferior endplate. Thus, regardless of changes in endplate pressure distribution, the sum of pressure converging on the waist remains the same and causes no bone reconstruction in this region. However, the above is only speculative and requires further validation by biomechanical studies.

Factors related to surgical manipulation may also contribute to ABL. The four endplates are subjected to surgical procedures such as burring. Due to their natural curvature, the endplates are prepared to better match the footprint. These insults could lead to bone remodeling and ABL. According to this explanation, bone resorption would not have occurred to the waist as it was barely surgically damaged, like what we observed.

It is worth noting that, as endplates untreated, group (2) exhibited opposed length changes to group (1). Possibly, this represented a continuation of natural degeneration accompanied by osteophytes formation at margin of the vertebral body [18]. Although surgery terminates the degeneration of operated segments, other segments remain exposed to factors associated with cervical spondylosis such as age-related changes, which may lead to continued degeneration [19].

### The particularity of middle vertebra

Middle vertebra had significant early area changes and happened more ABL. This could be due to its unique position: between two surgical segments, middle vertebra endured a “double” injury; clamped by two prostheses, middle vertebral body experienced a “double” bone reconstruction. However, rigorously, the intra-operative preparation of superior and inferior endplates is not identical [14], and the bone reconstruction may differ, too. Therefore, the change may not be a simple doubling. Besides, the endplates on middle vertebra themselves could be more prone to ABL in early post-operation. Opposed to the results of Kieser et al. [14], we detected differences in the incidence and degree of ABL in endplates with different positions (“between implants” or “not between implants” [14]), but the differences did not last beyond 6 months after surgery. The different results could be due to different prosthesis type, because Prestige LP was not included in their study.

The exception of middle vertebra deserves attention. To minimize the risk of complications, the ABL risk that middle vertebral body may suffer should be fully considered when planning multisegmental surgery and during intra-operative procedures, especially endplate milling and burring. Maximal preservation of middle vertebra volume can leave a buffer for post-operative ABL.

Lin et al. [20] observed collapse of the anterior edge of middle vertebral body in four patients after contiguous two-segmental ACDF with a zero-profile implant, while we did not observe a similar phenomenon in our study. They attributed the collapse to stress concentration and inner blood supply damage. The movable design of artificial disc may disperse the concentrated stress. In addition, in their study, four screws were inserted in middle vertebra. In contrast, the Prestige LP is stabilized by no screw but rails [21], which may cause less damage to the internal vertebral blood supply.

### Sagittal parameters: ROM, Cobb angle, T1S, and SVA

Overall and segment ROMs were well recovered and maintained. T1S varied between 22.53°±7.04° and 26.00°±6.37° over all follow-ups. SVA, although fluctuated, was not significantly changed, varying between 12.7 mm ± 6.9 and 15.0 mm ± 7.3 mm. These are consistent with our previous findings: CDA, although effective in preserving mobility, has a limited ability to improve sagittal alignment [22]. However, these parameters are still largely within the range to achieve good clinical outcomes according to previous studies. The review by Ling et al. suggested that the ranges are as follows: C7 or T1 slope with a mean value of 20° and no higher than 40°, cervical SVA with a mean value of 20 mm and no higher than 40 mm [23].

### Key pointsand limitations

The present study has the following key learning points. In contiguous two-level CDA, the middle vertebral body is special. More ABL happened to it, leading to detectable morphological changes on sagittal plain radiograph. In addition, this study proposed a new parameter, vertebral body sagittal area. Combined with anterior-posterior diameters and vertebral body heights, it helped to quantify morphological changes. For example, there has been no consensus on the method of comparing the degree of ABL per vertebra (not only per endplate), but in our study, the differences were detected successfully by sagittal area.

This study has the following limitations. Preoperative osteopenia was demonstrated to cause a higher ABL incidence [24], but the few osteopenia cases in our sample restricted further analysis. HO was assessed only on lateral radiographs, where there was some difficulty in identifying due to facet joint overlapping [25]. Furthermore, the conclusions drawn from this study are specific only to the Prestige LP artificial disc and may not be generalizable across other types. Finally, this study was a retrospective study with variable follow-up times.

## Conclusion

Sagittal area is a new imaging parameter that is easy to measure on lateral radiographs and helps to quantify morphological changes of vertebral body in sagittal plane. The decrease in sagittal area of middle vertebral body after contiguous two-segment CDA is more significant than adjacent vertebral bodies, which may be due to more anterior bone loss. The post-operative bone loss burden of middle vertebra should be fully considered during pre-operative planning and intra-operative manipulation to reduce the potential risk of prosthesis-related complications.

### Electronic supplementary material

Below is the link to the electronic supplementary material.

Supplementary Material 1

## Data Availability

The datasets generated during and/or analysed during the current study are available from the corresponding author on reasonable request.
