# Supplementary material for: More anterior bone loss in middle vertebra after contiguous two-segment cervical disc arthroplasty
Source: J Orthop Surg Res. 2024 Apr 12;19:234. doi: 10.1186/s13018-024-04663-6 (PMC11010387; doi:10.1186/s13018-024-04663-6)
Supplement: Supplementary file 1 — Supplementary Material 1 [file 13018_2024_4663_MOESM1_ESM.docx]

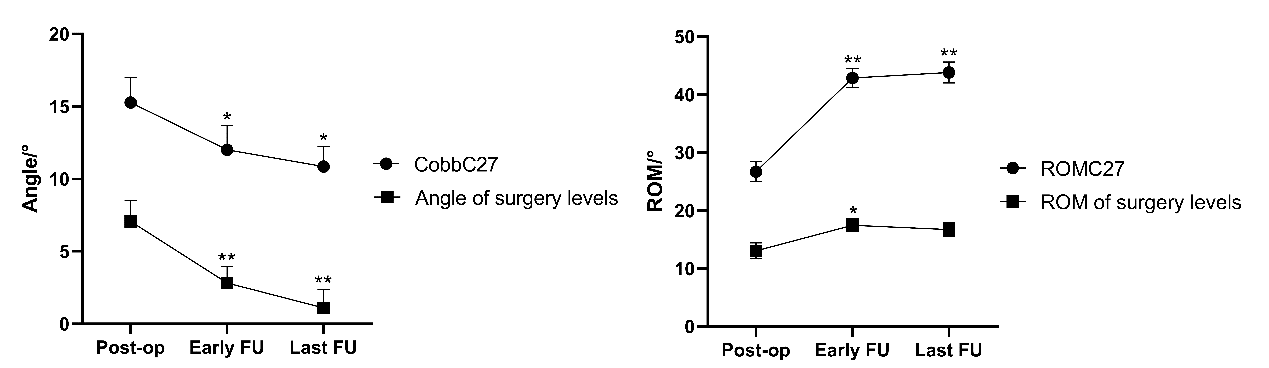


**Supplementary Fig.1** CobbC27 and angle of surgery levels, and ROMC27 and ROM of surgery levels after surgery

Post-op, post-operation; FU, follow-up; ROM, range of motion; *P<0.05, compared with post-operation in pairwise comparison;**P<0.001, compared with post-operation in pairwise comparison


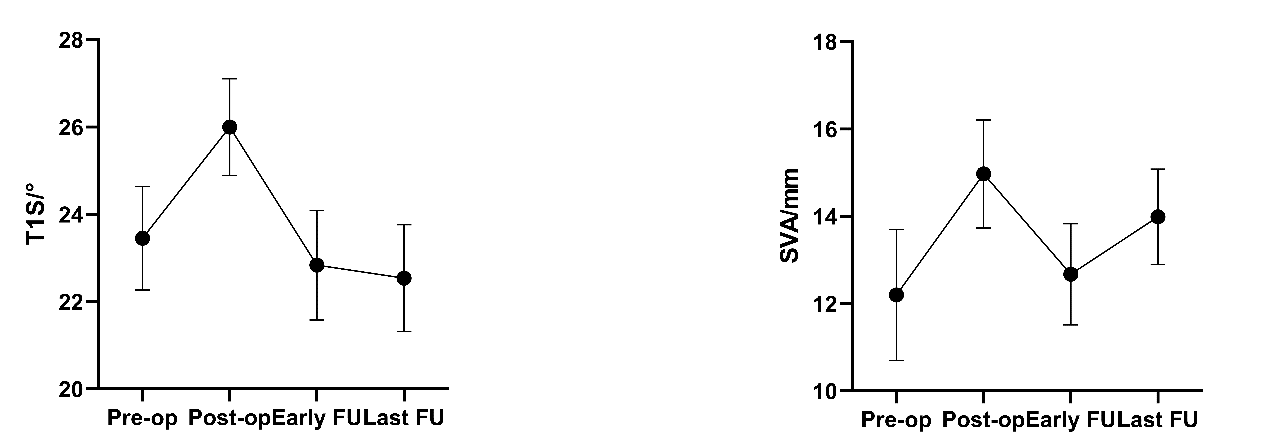


**Supplementary Fig.2** T1 slope and C2-C7 sagittal vertical axis before and after surgery

T1S, T1 slope; SVA, sagittal vertical axis; Pre-op, pre-operation; Post-op, post-operation; FU, follow-up.


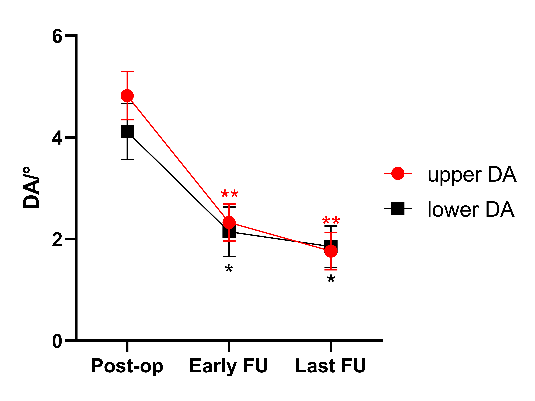


**Supplementary Fig.3** Disc angle after surgery

DA, disc angle; Post-op, post-operation; FU, follow-up. *P<0.05, compared with post-operation in pairwise comparison; **P<0.001, compared with post-operation in pairwise comparison

**Supplementary Table 1** Clinical outcomes scores at each follow-up

| Variables | Value |
| --- | --- |
| JOA scores |  |
| Pre-operative | 12.4±1.1 |
| Early FU | 15.4±0.8* |
| Last FU | 16.3±0.7* |
| NDI scores |  |
| Pre-operative | 27.2±1.8 |
| Early FU | 13.5±2.8* |
| Last FU | 6.4±2.8* |
| VAS scores |  |
| Pre-operative | 6.4±0.9 |
| Early FU | 2.9±0.7* |
| Last FU | 1.2±1.0* |

JOA, Japanese Orthopedic Association; NDI, Neck Disability Index; VAS, Visual analog scale; FU, follow-up. *P < 0.05, compared with pre-operation.

**Supplementary Table 2** HO incidence of segments

| FU period | Segment | Class of HO | | P |
| --- | --- | --- | --- | --- |
|  |  | Low degree | High degree |  |
| Early FU | Upper segment | 76(97.4%) | 2(2.6%) | <.001 |
|  | Lower segment | 72(92.3%) | 6(7.7%) | <.001 |
| Last FU | Upper segment | 56(71.8%) | 22(28.2%) |  |
|  | Lower segment | 54(69.2%) | 24(30.8%) |  |

HO, heterotopic ossification; FU, follow-up; P value is for comparison of early and late FU.

**Supplementary Table 3** ABL incidence and degree of the surgery-related endplates either on MV or not on MV

| FU | Endplate position | Degree of ABL | | | | P |
| --- | --- | --- | --- | --- | --- | --- |
| Period |  | No ABL | Mild ABL | Moderate ABL | Severe ABL |  |
| Early FU | On MV | 84(53.8%) | 24(15.4%) | 34(21.8%) | 14(8.0%) | 0.008 |
|  | Not on MV | 104(66.6%) | 28(19.0%) | 14(8.0%) | 10(6.4%) |  |
| Last FU | On MV | 86(55.1%) | 24(15.4%) | 32(20.5%) | 14(8.0%) | 0.175 |
|  | Not on MV | 96(61.5%) | 32(20.5%) | 10(6.4%) | 18(11.5%) |  |

FU, follow-up; ABL, anterior bone loss; MV, middle vertebra; P is for comparison between endplates on MV or not at each follow-up period.
